# The association between estimated glucose disposal rate and self-reported diabetic retinopathy: evidence from two independent cohorts and machine learning

**DOI:** 10.3389/fendo.2026.1853329

**Published:** 2026-06-26

**Authors:** Chaofeng Yuan, Yue Hao, Jianghui Wang, Chuanxi Wang, Zhengxuan Jiang

**Affiliations:** 1 Department of Ophthalmology, The Second Affiliated Hospital of Anhui Medical University, Anhui Medical University, Hefei, Anhui, China; 2 Department of Ophthalmology, The first Affiliated Hospital of Zhengzhou University, Zhengzhou University, Zhengzhou, Henan, China

**Keywords:** cross-sectional study, diabetic retinopathy, eGDR, insulin resistance, machine learning

## Abstract

**Background:**

Insulin resistance plays a key role in the pathogenesis of diabetic retinopathy (DR). Although established insulin resistance markers have been shown to predict a variety of complications, the association between the estimated glucose disposal rate (eGDR) and prevalence of DR remains incompletely characterized. This study aims to examine the relationship between eGDR and DR prevalence.

**Methods:**

This cross-sectional study analyzed complete participant data (N = 1, 536) from the 2007–2018 National Health and Nutrition Examination Survey (NHANES) for all relevant information. The relationship between the insulin resistance index and self-reported DR prevalence was evaluated by using multivariate logistic regression and a restricted cubic spline (RCS) model. Subgroup analysis was conducted to assess heterogeneity across groups, and two sensitivity analyses were performed to assess the robustness of the results. In machine learning, the Boruta algorithm is applied for feature selection. The selected features are subsequently utilized by XGBoost and random forest models for DR prevalence estimation. Use the Shapley additive explanations (SHAP) value to explain the independent contribution of eGDR. In the clinical cohort, we recruited patients who visited the Second Affiliated Hospital of Anhui Medical University from September 1, 2025, to December 30, 2025. A total of 297 participants who met the inclusion criteria were finally enrolled. Multivariable logistic regression and RCS curves were used to validate the findings from the NHANES analysis.

**Results:**

In the fully adjusted model, eGDR and self-reported DR prevalence show a significant negative linear correlation (OR = 0.79, 95% CI: 0.67–0.93, P = 0.0049). Subgroup and sensitivity analyses confirm the stability of this negative association. The Boruta algorithm identifies eGDR as a robust and important feature. Both the XGBoost (AUC = 0.773) and random forest (AUC = 0.764) models show moderate predictive performance, and eGDR has high variable importance. SHAP analysis indicates that eGDR, together with body mass index and income poverty, is a key determinant of self-reported DR prevalence. The results of the clinical cohort are like NHANES.

**Conclusion:**

This cross-sectional study suggested that lower eGDR is associated with a higher prevalence of self-reported DR. Accordingly, eGDR may serve as a potential marker for risk stratification rather than a causal or preventive factor. Prospective longitudinal research is necessary to confirm these findings and to explore whether a causal relationship exists.

## 1 Introduction

Diabetic retinopathy (DR) is the most common microvascular complication of diabetes. It is characterized by a series of pathological changes, including retinal microvascular leakage, capillary ischemia, and pathological neovascularization, which eventually lead to progressive vision damage or irreversible blindness (1, 2). According to the Diabetes Atlas, approximately 160 million individuals with diabetes worldwide are affected by DR (3). Three interrelated global trends cause this heavy burden of disease: the rapid rise in the prevalence of diabetes, the accelerated aging of the population, and the persistent low rate of primary health-based eye screening (4, 5). Although intravitreal injection of anti-vascular endothelial growth factor (anti-VEGF) therapy has been proven to delay vision loss, its high economic burden and limited therapeutic effect significantly limit its impact at the population level (6, 7).

The synergistic effect of insulin resistance (IR) and chronic hyperglycemia leads to progressive damage to retinal microvasculature (8, 9). Traditional metabolic markers, such as glycated hemoglobin (HbA1c) and fasting blood glucose (FPG), mainly reflect average or single blood sugar levels (10, 11). These indicators are insufficient to capture short-term fluctuations in blood glucose, especially for the dynamic assessment of insulin resistance (12). To address these limitations, this study adopts a multidimensional, complex insulin resistance index. Specifically, the steady-state model evaluates the insulin resistance index (HOMA-IR) based on the dynamic changes of glucose-insulin to evaluate fasting insulin resistance (13); HOMA-β evaluates the secretion capacity and compensatory function of pancreatic beta cells under hyperglycemia conditions (14); HOMA-IS quantifies peripheral Insulin sensitivity, which reflects the efficiency of glucose uptake at the tissue level (15). In addition, the insulin resistance metabolism score (METS-IR) integrates the indicators of visceral fat and dyslipidemia, including waist circumference, triglycerides (TG), fasting blood glucose (FPG), and high-density lipoprotein cholesterol (HDL-C) (16); the estimated glucose disposal rate (eGDR) dynamically reflects the coupling relationship between tissue glucose utilization capacity and metabolic function and vascular function (17). Collectively, this multidimensional framework overcomes the inherent limitations of traditional glycemic indices and provides a novel paradigm for the precise prevention and control of IR-driven microvascular injury (18).

As a core pathogenic mechanism of DR, insulin resistance has been shown to correlate with various ophthalmic evaluation indicators (19, 20). Early studies using single insulin resistance indicators (such as HOMA-IR) found a significant correlation between basal insulin sensitivity and DR prevalence (21). Subsequently, Bao and colleagues further confirmed that insulin resistance was positively correlated with DR progression (22). In addition, the clinical value of insulin resistance-related indicators has been widely demonstrated across studies on metabolic disorders and eye diseases (23). However, the accumulation of this evidence and the systematic evaluation of the relationship between multidimensional, comprehensive indicators of insulin resistance and DR remain limited (24, 25).

Against this background, this study aims to explore the relationship between multiple indicators of complex insulin resistance and the prevalence of self-reported DR using data from the National Health and Nutrition Examination Survey (NHANES). This analysis presents a more comprehensive metabolic view of the association between eGDR and self-reported DR prevalence and may provide a useful foundation for future longitudinal research on the role of metabolic factors in DR.

## 2 Methods

### 2.1 Study design and population

The NHANES is an ongoing, biennial, nationally representative cross-sectional survey designed to assess the health and nutritional status of the civilian population in the United States. The Institutional Review Board of the United States National Center for Health Statistics has approved the NHANES program. It has obtained written informed consent from all participants before data collection.

Participants were identified as having diabetes based on any of the following criteria: (1) fasting glucose ≥7.0 mmol/L, (2) glycated hemoglobin (HbA1c) ≥6.5%, (3) current use of antidiabetic medications, or (4) self-reported physician diagnosis of diabetes.

**Figure 1:**
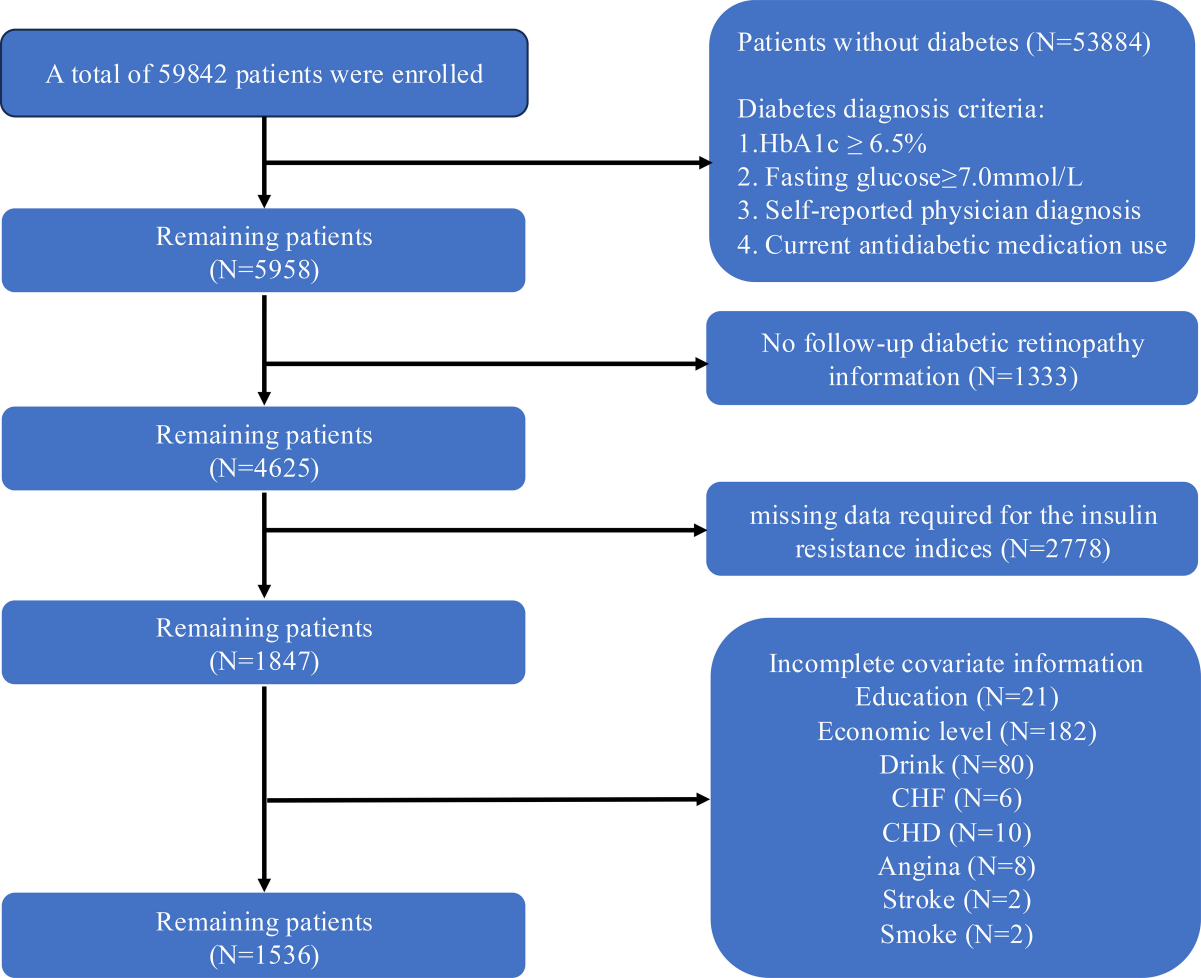
Flowchart of participants selection and grouping.

In this study, we analyzed data from 2007 to 2018, including 59, 842 participants. Participants are excluded according to the following criteria: (1) participants without diabetes (N = 53884); (2) lack of self-reported DR assessment data (n = 1333); (3) lack of data required to calculate the insulin resistance index (n = 2, 778); (4) lack of relevant covariable information (n = 312). After applying these exclusion criteria, the final analysis sample included 1, 536 participants. The detailed flow chart is shown in **Figure 1**.

We recruited patients with DR who visited the Ophthalmology Outpatient Clinic at the Second Affiliated Hospital of Anhui Medical University between September 1, 2025, and December 30, 2025. Inclusion criteria: 1) Age over 18 years; 2) Underwent a fundus examination and received a definitive diagnosis of diabetic retinopathy from an associate chief physician or chief physician; 3) Complete demographic data (age, sex, education level, height, weight, waist circumference), HbA1c levels, and information about hypertension and cardiac health. Exclusion criteria: 1) Presence of severe systemic diseases; 2) Pregnancy or lactation. The control group included diabetic patients who underwent health examinations at the screening center during the same recruitment period. All control participants were confirmed free of DR and other ocular diseases by comprehensive fundus examination. The inclusion and exclusion criteria were consistent with those applied to the case group as described above. Ultimately, a total of 259 participants were enrolled in this clinical cohort. This study was approved by the hospital’s ethics committee. (YX2023-142(F1)).

### 2.2 Assessment of self-reported diabetic retinopathy

The diagnosis of DR was obtained from participants’ responses to the questionnaire item: “ Has diabetes affected your eyes, or have you ever had retinopathy?” Participants who answered “yes” were classified as DR patients. This self-reported assessment method was widely used in many studies on diabetes complications based on NHANES, and it is practical and satisfactory (26, 27).

### 2.3 Calculation of insulin resistance indicators

We use fasting insulin, fasting blood glucose, triglycerides, body mass index (BMI), high-density lipoprotein cholesterol (HDL-C), waist circumference, hypertension status, and glycated hemoglobin (HbA1c) to calculate multiple indices of insulin resistance. The unit of BMI is kilograms/square meter (kg/m²), and the unit of waist circumference is centimeters (cm). Hypertension is regarded as a binary variable. The detailed calculation formula is provided in **Supplementary Table 1**.

### 2.4 Covariates

Based on prior literature and biological rationale, this study incorporates a set of comprehensive covariates into the analysis. These variables include demographic characteristics, BMI, health-related behaviors, systemic complications, and a history of cardiovascular disease. The detailed definitions and classifications of all covariates are provided in **Supplementary Table 2**.

In the externally validated clinical cohort, we included baseline demographic variables (age, gender, education level, height, weight, and waist circumference), smoking and drinking habits, HbA1c levels, hypertension status, and cardiovascular disease.

### 2.5 Statistical analysis

NHANES employs a complex, multistage, stratified probability sampling design. This study collected data from six consecutive NHANES cycles spanning 12 years (2007-2018). To obtain nationally representative estimates, we incorporated the mobile examination center (MEC) examination weights, along with stratification variables (SDMVSTRA) and primary sampling units (SDMVPSU). For each 2-year cycle, the corresponding 2-year MEC examination weight (WTMEC2YR) was used. To combine the six cycles, a 12-year pooled MEC weight (WTMEC12YR) was constructed according to NCHS analytic guidelines using the following formula:


WTMEC12YR = (1/6) × WTMEC2YR

This standardization ensures that each 2-year cycle contributes equally to the pooled estimates. The survey package was used to account for the complex survey design. Continuous variables are represented by weighted means and standard deviation, while categorical variables are expressed as unweighted sample sizes and weighted percentages.

Construct a weighted logistic regression model to evaluate the relationship between insulin resistance indicators and self-reported DR prevalence. Model 1: unadjusted; Model 2: adjusted for age, gender, and race; Model 3 further adjusted for all covariates. Subsequently, the restricted cubic spline (RCS) model was used to test the nonlinear association between the insulin resistance index and self-reported DR prevalence. We applied 4 knots at the 5th, 35th, 65th, and 95th percentiles of eGDR, with the median value used as the reference.

A subgroup analysis was carried out by stratification by age, gender, ethnicity, education level, smoking status, and alcohol consumption, and interaction terms were tested to assess the consistency of the association across subgroups. The receiver operating characteristic curve (ROC) curve was constructed to analyze and evaluate the predictive performance of insulin resistance indicators, using the area under the curve (AUC) as a measure of discrimination. Two sensitivity analyses were carried out to evaluate the robustness of the research results: (1) excluding participants with extreme (<2 or ≥8) values of eGDR; (2) analysis without applying sampling weights. In the external validation cohort, we used multivariate logistic regression and RCS curves to validate our findings.

In addition, machine learning approaches, including Random Forest and Extreme Gradient Boosting (XGBoost), were employed to evaluate variable importance for predicting the prevalence of DR. To minimize the risk of data leakage, Boruta feature selection was performed solely within the training set after data splitting. Participants were randomly divided into a training set (70%) and a validation set (30%) using stratified random sampling to maintain the original class distribution of DR. No cross-validation was used for the final model evaluation; instead, model performance was assessed on the independent validation set. Hyperparameters were tuned using grid search with 5-fold cross-validation within the training set. Given the class imbalance in the outcome, the Synthetic Minority Over-sampling Technique (SMOTE) was applied in the training set to address this issue. Model calibration was evaluated using the Brier score and calibration plots. After feature selection and hyperparameter tuning, the final models were trained on the training set and evaluated on the validation set. SHAP values were calculated to quantify the contribution of each variable to the model predictions. Statistical significance is defined as a bilateral P-value < 0.05. All analyses are carried out using R version 4.3.1.

## 3 Results

### 3.1 Baseline characteristics

A total of 1536 participants were included in this study, of which 818 (52.6%) were male. The average age is 59.84 years old. Of all participants, 298 were diagnosed with self-reported DR. **Table 1** summarizes baseline sociodemographic and clinical characteristics stratified by self-reported DR status. Individuals with self-reported DR are more likely to be male and non-Hispanic blacks or non-Hispanic whites. In addition, participants with self-reported DR are usually less educated, have a moderate family income, are more likely to be married or live together, and have a history of drinking. From a clinical perspective, the prevalence of complications in the DR group was higher, including obesity and hypertension.

**Table 1:** Demographics classified with self-reported DR.

Variables	All	Non-self-reported DR	Self-reported DR	p-value
Number	1536	1238	298	
Gender (N, %)				0.709
Male	818 (52.6)	649 (52.3)	169 (54.4)	
Female	718 (47.4)	589 (47.7)	129 (45.6)	
Age [years, mean (SD)]	59.84 (12.96)	59.77 (12.98)	60.21 (12.90)	0.644
Ethnicity (N, %)				0.059
Mexican American	263 (8.8)	221 (9.0)	42 (7.8)	
Other Hispanic	185 (6.3)	146 (6.2)	39 (6.8)	
Non-Hispanic White	575 (64.4)	478 (65.6)	97 (58.5)	
Non-Hispanic Black	366 (13.0)	286 (12.3)	80 (16.5)	
Other	147 (7.5)	107 (6.9)	40 (10.5)	
Education (N, %)				0.018
Less Than 9th Grade	240 (9.1)	195 (8.7)	45 (11.0)	
9-11th Grade	271 (13.7)	209 (12.3)	62 (20.6)	
Highschool graduate or equivalent	339 (24.9)	274 (24.2)	65 (28.3)	
Some Colleges or AA degree	429 (30.9)	348 (32.1)	81 (25.1)	
College graduate or above	257 (21.4)	212 (22.8)	45 (14.9)	
BMI				0.793
<25	194 (10.4)	158 ( (10.6)	36 (9.6)	
[25, 30]	467 (27.5)	375 (27.2)	92 (29.3)	
>30	875 (62.0)	705 (62.2)	170 (61.1)	
Economic level (N, %)				0.013
<1	341 (14.9)	264 (13.8)	77 (20.2)	
1-3	734 (42.9)	589 (41.6)	145 (48.9)	
>3	461 (42.2)	385 (44.6)	76 (31.0)	
Marital status (N, %)				0.742
Unmarried or other	614 (35.6)	493 (35.8)	121 (34.4)	
Married or living with a partner	922 (64.4)	745 (64.2)	177 (65.6)	
Alcohol consumption (N, %)				0.136
Yes	1077 (71.9)	871 (73.1)	206 (65.9)	
No	459 (28.1)	367 (26.9)	92 (34.1)	
Smoking status (N, %)				0.404
Never	767 (50.2)	609 (49.1)	158 (55.1)	
Now	237 (14.5)	194 (14.5)	43 (14.1)	
Former	532 (35.4)	435 (36.4)	97 (30.8)	
Hypertension (N, %)				0.094
Yes	1143 (72.6)	905 (71.4)	238 (78.4)	
No	393 (27.4)	333 (28.6)	60 (21.6)	
CHD (N, %)				0.137
Yes	175 (11.4)	129 (10.6)	46 (15.4)	
No	1361 (88.6)	1109 (89.4)	252 (84.6)	
CHF (N, %)				0.001
Yes	143 (8.7)	91 (7.0)	52 (16.6)	
No	1393 (91.3)	1147 (93.0)	246 (83.4)	
Angina (N, %)				0.660
Yes	107 (7.4)	78 (7.2)	29 (8.4)	
No	1429 (92.6)	1160 (92.8)	269 (91.6)	
Stroke (N, %)				<0.001
Yes	139 (8.0)	92 (6.3)	47 (16.0)	
No	1397 (92.0)	1146 (93.7)	251 (84.0)	
HOMA_IR (mean (SD))	10.72 (14.53)	10.04 (12.29)	13.99 (22.05)	0.062
HOMA_IS (mean (SD))	0.28 (0.62)	0.28 (0.64)	0.31 (0.52)	0.467
HOMA_B (mean (SD))	137.35 (495.60)	140.88 (413.65)	120.51 (776.38)	0.615
METS_IR (mean (SD))	57.45 (18.51)	57.43 (18.51)	57.52 (18.53)	0.953
eGDR (mean (SD))	4.56 (2.48)	4.65 (2.48)	4.08 (2.42)	0.015
QUICKI (mean (SD))	2.88 (3.00)	3.00 (0.97)	2.30 (6.86)	0.402

Continuous variables are presented as weighted means (standard Deviation). Categorical variables are presented as unweighted counts (weighted percentages).

DR, diabetic retinopathy; SD, Standard Deviation; CHD, Coronary heart disease; CHF, congestive heart failure.

### 3.2 Multivariable logistic regression and restricted cubic spline analyses

**Figure 2:**
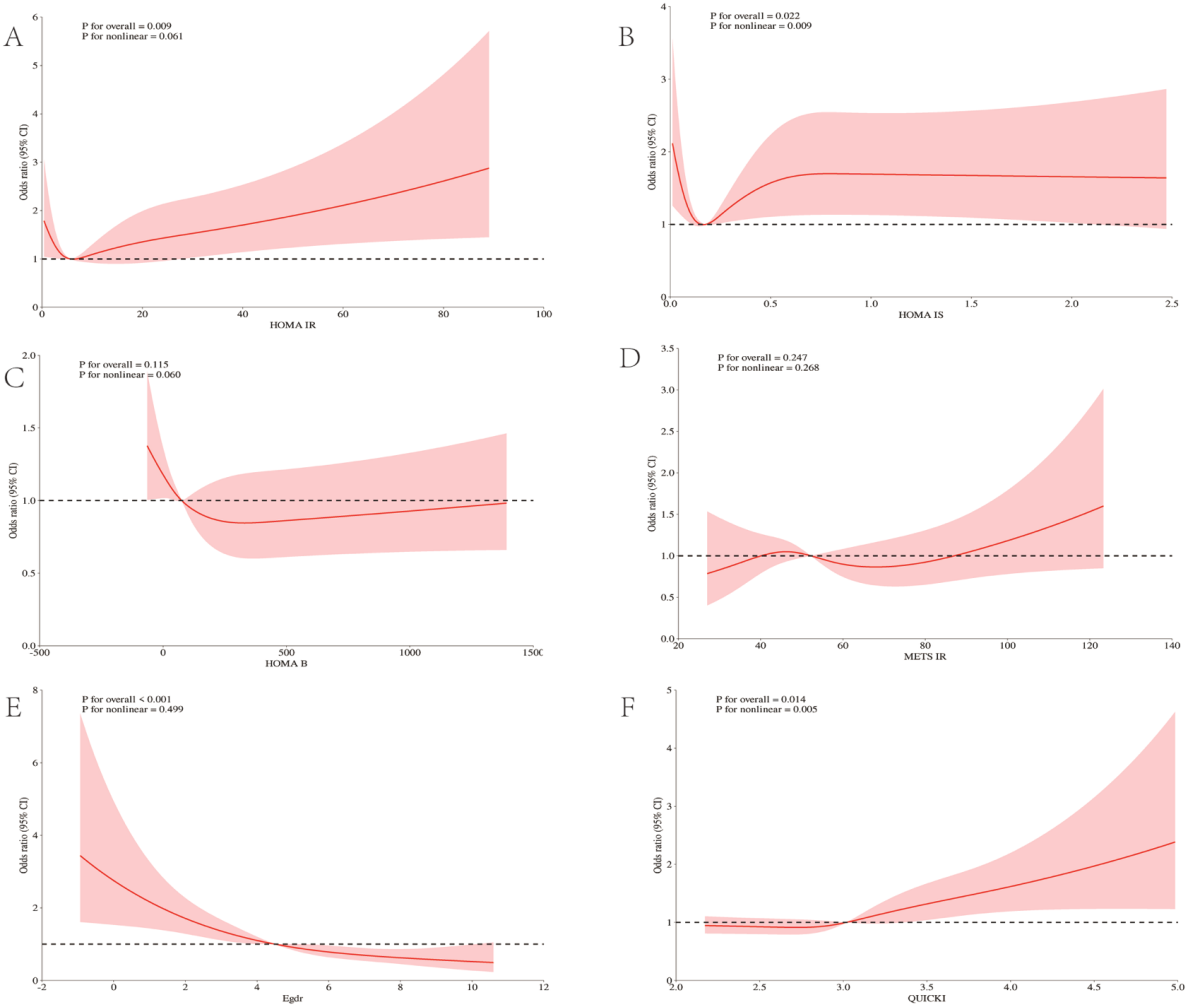
Restricted cubic spline models illustrating the dose-response relationships between various insulin resistance-related indices and self-reported DR. The red line represents the estimated odds ratio, and the pink shaded area represents the 95% confidence interval. P values indicate the significance of the overall association and nonlinearity. All models were adjusted for all covariates. **(A)** HOMA-IR; **(B)** HOMA-IS; **(C)** HOMA-B; **(D)** METS-IR; **(E)** eGDR; **(F)** QUICKI.

We conducted a multivariate weighted logistic regression analysis to evaluate the relationship between multiple insulin resistance indicators and the prevalence of self-reported DR (**Table 2**). In model 3, for each additional unit of eGDR, the prevalence of self-reported DR is reduced by 21% (OR = 0.79, 95% CI: 0.67–0.93, P = 0.0049). When eGDR is divided into quartiles, in model 3, the prevalence of self-reported DR is significantly lower than in the lowest quartile (OR = 0.31, 95% CI: 0.13–0.75, P = 0.0105). These results consistently show that there is a significant negative correlation between eGDR and self-reported DR prevalence. The results of RCS show that significant nonlinear correlations were observed in HOMA-IS (nonlinear P value = 0.009) and QUICKI (nonlinear P value = 0.005). In contrast, no nonlinear relationship was detected between eGDR and self-reported DR prevalence (nonlinear P value = 0.499), suggesting a roughly linear negative correlation between the two (**Figure 2**).

**Table 2:** The relationship between IR indicators and the prevalence of self-reported DR.

Variable	Model 1		Model 2		Model 3	
	OR (95%CI)	p-value	OR (95%CI)	p-value	OR (95%CI)	p-value
HOMA_IR (continuous)	1.01 (1.00, 1.03)	0.0188	1.02 (1.00, 1.03)	0.0174	1.02 (1.00, 1.03)	0.0126
HOMA_IR (multi-category)						
Q1	ref	ref	ref	ref	ref	ref
Q2	0.77 (0.45, 1.29)	0.3132	0.79 (0.46, 1.35)	0.3748	0.76 (0.45, 1.28)	0.3022
Q3	0.61 (0.35, 1.06)	0.0764	0.63 (0.35, 1.13)	0.1199	0.59 (0.40, 1.01)	0.0528
Q4	0.98 (0.62, 1.57)	0.9445	1.04 (0.64, 1.68)	0.8664	0.99 (0.64, 1.53)	0.9579
The relationship between HOMA_IS and prevalence of DR						
HOMA_IS (continuous)	1.07 (0.88, 1.31)	0.4850	1.08 (0.88, 1.31)	0.4608	1.06 (0.88, 1.27)	0.5630
HOMA_IS (multi-category)						
Q1	ref	ref	ref	ref	ref	ref
Q2	0.64 (0.37, 1.11)	0.1080	0.63 (0.37, 1.07)	0.0859	0.62 (0.38, 1.01)	0.0539
Q3	0.79 (0.45, 1.38)	0.4000	0.76 (0.43, 1.37)	0.3589	0.78 (0.45, 1.35)	0.3736
Q4	1.03 (0.64, 1.64)	0.9030	0.97 (0.60, 1.58)	0.9095	1.03 (0.66, 1.59)	0.9044
The relationship between HOMA_B and prevalence of DR						
HOMA_B (continuous)	0.99 (0.99, 1.00)	0.5430	0.99 (0.99, 1.00)	0.4922	0.99 (0.99, 1.00)	0.5383
HOMA_B (multi-category)						
Q1	ref	ref	ref	ref	ref	ref
Q2	0.70 (0.40, 1.23)	0.2066	0.70 (0.38, 1.27)	0.2361	0.72 (0.37, 1.41)	0.3357
Q3	0.40 (0.23, 0.73)	0.0028	0.41 (0.22, 0.78)	0.0066	0.40 (0.20, 0.80)	0.0103
Q4	0.69 (0.41, 1.16)	0.1564	0.70 (0.40, 1.22)	0.2028	0.66 (0.36, 1.21)	0.1789
The relationship between METS_IR and prevalence of DR						
METS_IR (continuous)	1.00 (0.99, 1.01)	0.9520	1.00 (0.99, 1.01)	0.7312	1.00 (0.99, 1.01)	0.8091
METS_IR (multi-category)						
Q1	ref	ref	ref	ref	ref	ref
Q2	0.98 (0.57, 1.68)	0.9480	1.05 (0.62, 1.78)	0.8652	1.08 (0.62, 1.87)	0.7930
Q3	0.99 (0.56, 1.78)	0.9930	1.07 (0.58, 1.96)	0.8250	0.99 (0.52, 1.90)	0.9901
Q4	0.89 (0.53, 1.48)	0.6400	0.97 (0.56, 1.68)	0.8984	0.93 (0.50, 1.74)	0.8281
The relationship between eGDR and prevalence of DR						
eGDR (continuous)	0.91 (0.84, 0.99)	0.0205	0.91 (0.83, 0.99)	0.0294	0.79 (0.67, 0.93)	0.0049
eGDR (multi-category)						
Q1	ref	ref	ref	ref	ref	ref
Q2	0.48 (0.26, 0.86)	0.0146	0.46 (0.24, 0.88)	0.0189	0.37 (0.19, 0.73)	0.0051
Q3	0.63 (0.33, 1.21)	0.1625	0.62 (0.30, 1.27)	0.1880	0.42 (0.20, 0.91)	0.0284
Q4	0.50 (0.30, 0.84)	0.0090	0.49 (0.29, 0.85)	0.0115	0.31 (0.13, 0.75)	0.0105
The relationship between QUICKI and prevalence of DR						
QUICKI (continuous)	0.95 (0.91, 0.99)	0.0153	0.94 (0.90, 0.99)	0.0110	0.95 (0.91, 0.98)	0.0118
QUICKI (multi-category)						
Q1	ref	ref	ref	ref	ref	ref
Q2	0.61 (0.37, 1.03)	0.0638	0.62 (0.37, 1.04)	0.0713	0.62 (0.37, 1.04)	0.0710
Q3	1.16 (0.72, 1.86)	0.5293	1.14 (0.71, 1.82)	0.5777	1.18 (0.74, 1.90)	0.4860
Q4	1.33 (0.78, 2.28)	0.2918	1.29 (0.73, 2.28)	0.3688	1.36 (0.75, 2.49)	0.3074

Model 1: unadjusted.

Model 2: Model 1 + sex, age, and ethnicity.

Model 3: Model 2 + educational level, marital status, economic level, BMI, alcohol consumption, smoking status, hypertension, CHF, CHD, angina, and stroke.

BMI, body mass index; OR, odds ratio; CI, confidence interval; eGDR, estimated glucose disposal rate; DR, diabetic retinopathy.

### 3.3 Subgroup analyses

**Figure 3:**
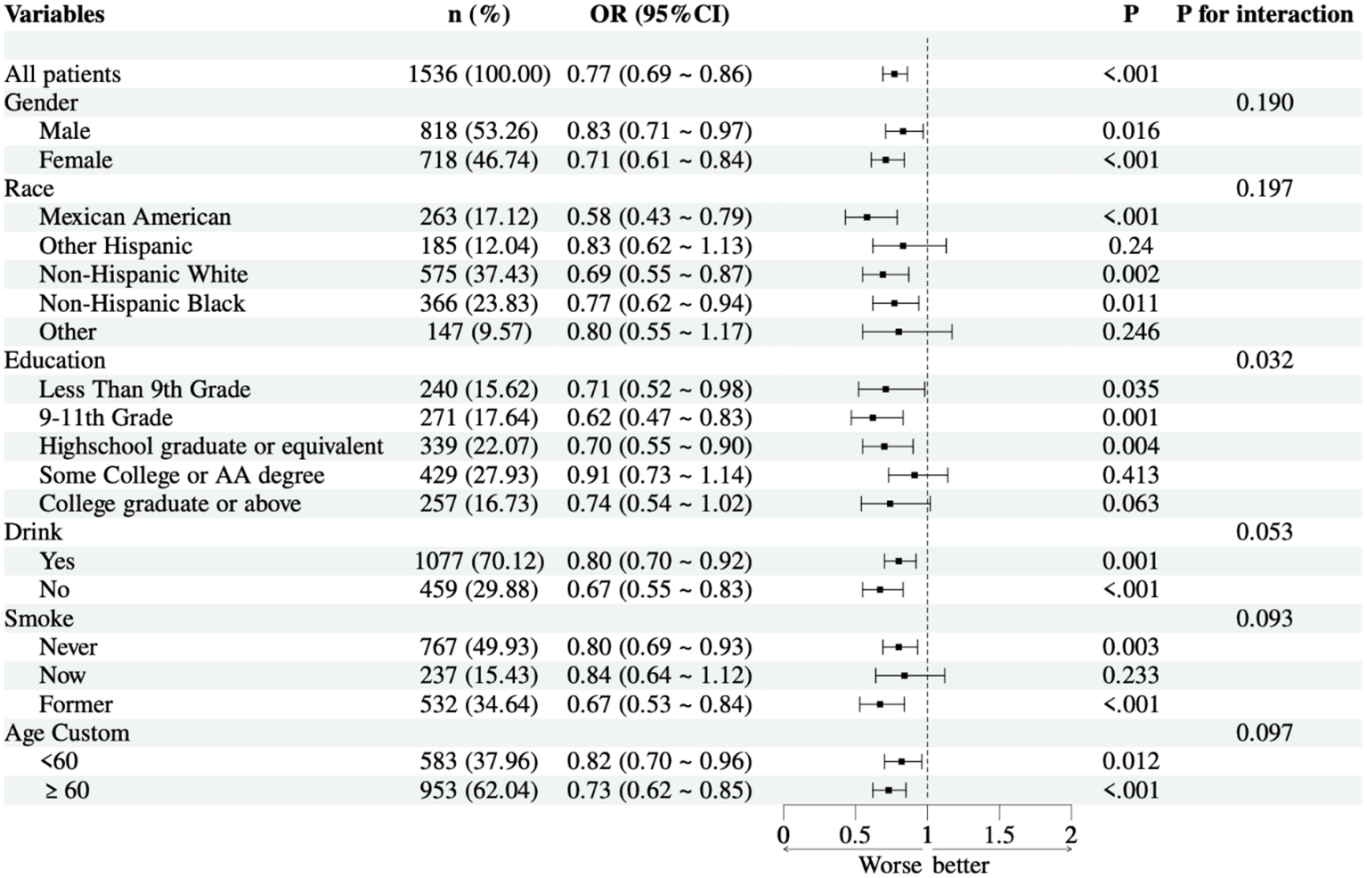
Stratified analysis on the association between eGDR and self-reported DR. Adjusted for all covariates.

We conducted a subgroup analysis to assess the consistency of the association between eGDR and self-reported DR prevalence across the predefined population stratifications. (**Figure 3**) A negative correlation between eGDR and self-reported DR prevalence was observed across all subgroups. It is worth noting that this negative correlation is more pronounced among women and non-drinkers. In these subgroups, for each additional unit of eGDR, the prevalence of self-reported DR in women is reduced by 29% (OR = 0.71, 95% CI: 0.61–0.84), and the prevalence of self-reported DR in non-drinkers is reduced by 33% (OR = 0.67, 95% CI: 0.55–0.83).

### 3.4 Sensitivity analyses

To evaluate the robustness of the main research results, we conducted two sensitivity analyses (**Supplementary Tables 4**, **S5**). First, after excluding participants with extreme eGDR values, the negative correlation between eGDR and self-reported DR prevalence is still statistically significant (OR = 0.76, 95% CI: 0.60–0.97, P = 0.0250). Secondly, the analysis using unweighted data also obtained the same results (OR = 0.77, 95% CI: 0.60–0.97, P <0.0001), further supporting the stability and reliability of the observed association.

### 3.5 Clinical study results

**Figure 4:**
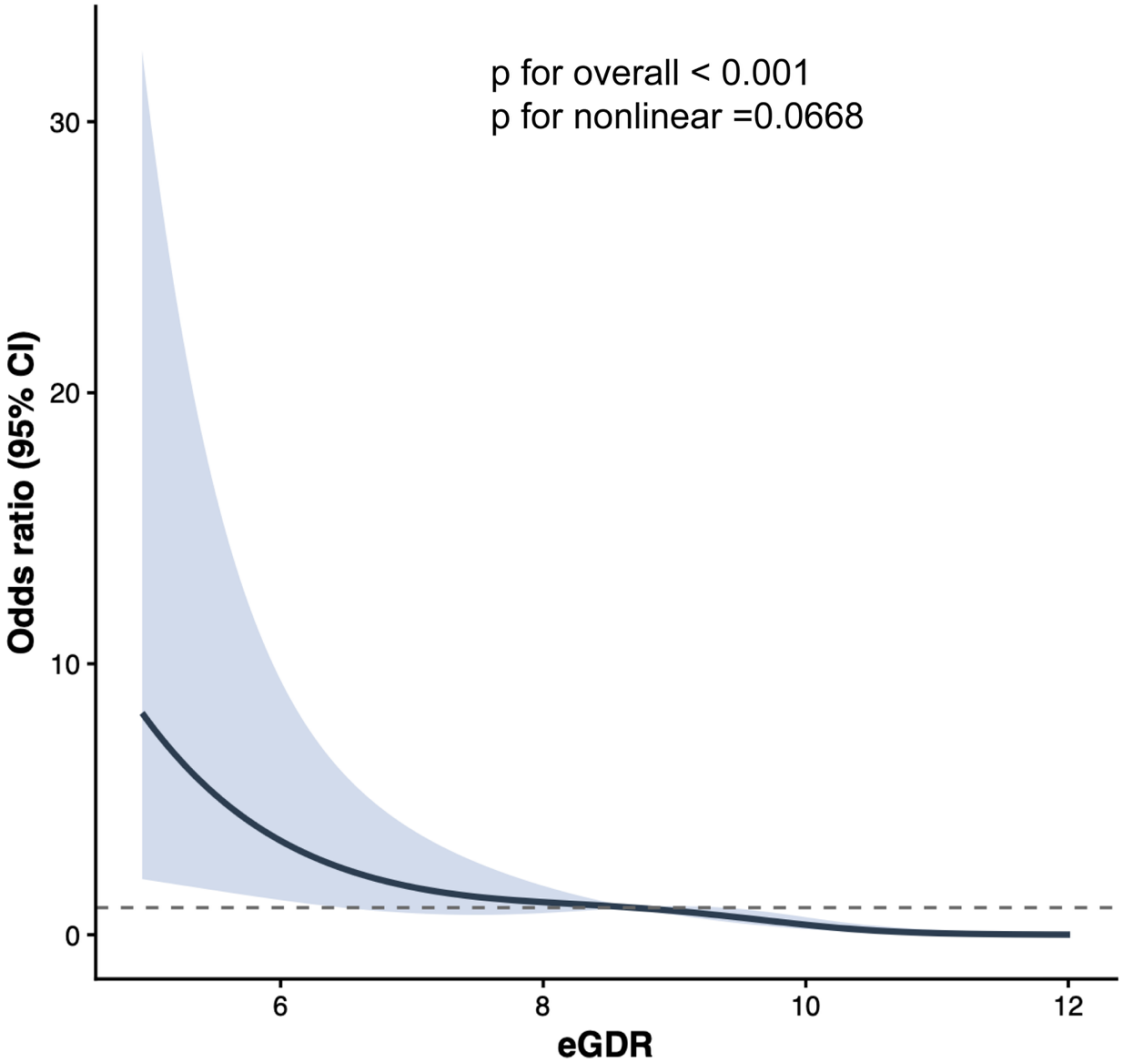
RCS of eGDR and prevalence of DR in the clinical study. The black line represents the estimated odds ratio, and the blue shaded area represents the 95% confidence interval. The model was adjusted for all covariates.

To validate our findings, we established a Chinese population-based cohort from the Second Affiliated Hospital of Anhui Medical University to investigate the association between eGDR, and the prevalence of DR. Baseline table results indicate that, compared with the group without DR, the eGDR was significantly lower in the group with DR (**Table 3**). Results from multivariable logistic regression, after fully adjusting for covariates, showed that eGDR was negatively associated with the prevalence of DR [OR = 0.44, 95% CI: (0.32–0.57), P < 0.001] (**Table 4**). RCS results indicated a linear negative correlation between eGDR and DR prevalence (**Figure 4**). These findings are consistent with conclusions drawn from the NHANES database.

**Table 3:** Characteristics of participants stratified by DR from the clinical study.

Variables	All	Non-DR	DR	p-value
Number	259	152	107	
Gender (N, %)				0.899
Male	152 (58.7)	89(58.6)	63(58.9)	
Female	107 (41.3)	63(41.4)	44(44.1)	
Age [years, mean (SD)]	51.49 (14.19)	48.20 (14.09)	56.17 (13.03)	<0.001
Education (N, %)				0.348
**Illiterate**	29 (11.2)	17 (11.2)	12 (11.2)	
Primary school	65 (25.1)	44 (28.9)	21 (19.6)	
Middle school	90 (34.7)	48 (31.6)	42 (39.3)	
High school and above	75 (29.0)	43 (28.3)	32 (29.9)	
BMI (kg/m2)	24.95 (4.13)	24.75 (3.81)	25.25 (4.56)	0.343
Alcohol consumption (N, %)				0.290
Never	206 (79.5)	116 (76.3)	90 (84.1)	
Rarely	42 (16.2)	28 (18.4)	14 (13.1)	
Often	11 (4.2)	8 (5.3)	3 (2.8)	
Smoking status (N, %)				0.934
No	210 (81.1)	124 (81.6)	86 (80.4)	
Yes	49 (18.9)	28 (18.4)	21 (19.6)	
Hypertension (N, %)				0.007
Yes	102 (39.4)	49 (32.2)	53 (49.5)	
No	157 (60.6)	103 (67.8)	54 (50.5)	
CHD (N, %)				0.009
Yes	31 (12.0)	11 (7.2)	20 (18.7)	
No	228 (88.0)	141 (92.8)	87 (81.3)	
eGDR	8.61 (2.14)	9.25 (2.06)	7.71 (1.93)	<0.001

SD, Standard Deviation; BMI, body mass index; CHD, coronary heart disease. lliterate: refers to participants who are unable to read and write.

**Table 4:** Multivariate logistic regression analysis of eGDR and prevalence of DR in a clinical study.

Variables	Model 1	Model 2	Model 3
	OR (95% CI) P-value	OR (95% CI) P-value	OR (95% CI) P-value
eGDR	0.70(0.61, 0.79), <0.001	0.73(0.63, 0.83), <0.001	0.44(0.32, 0.57), <0.001

Model 1: unadjusted.

Model 2: Model 1+age and gender.

Model 3: Model2 + educational level, BMI, smoking status, alcohol consumption, hypertension, and CHD.

BMI, body mass index; OR, odds ratio; CI, confidence interval; CHD, Coronary heart disease.

### 3.6 Machine learning results

**Figure 5:**
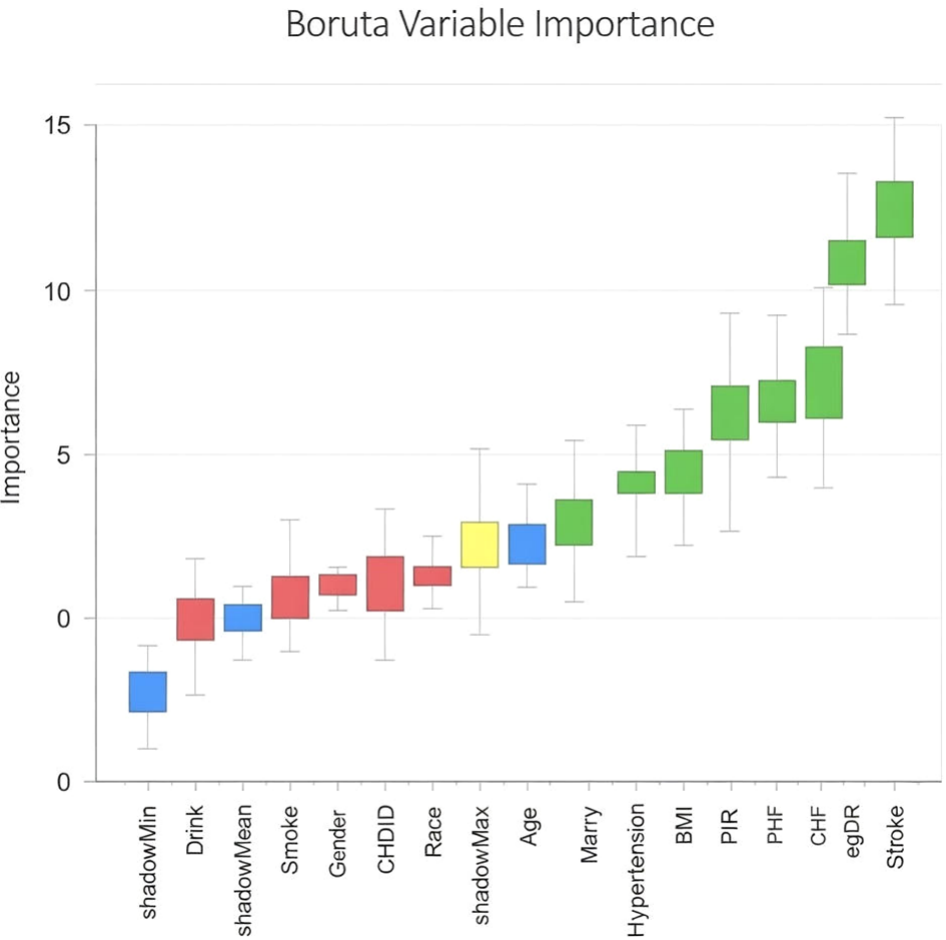
Boruta algorithm ranking of key risk factors for self-reported DR prevalence. Blue: shadow; red: rejected; yellow: tendensive; green: confirmed.

To identify the important characteristics associated with self-reported DR prevalence, we first use the Boruta algorithm to evaluate the importance of all candidate variables (**Figure 5**). The Boruta method determines feature correlations by comparing the original variable’s importance with that of a randomly permuted “shadow” variable to identify predictors that contribute stably to the results. Among all the evaluated characteristics, eGDR ranks significantly higher and is much more important than most clinical, metabolic, and sociodemographic variables.

**Figure 6:**
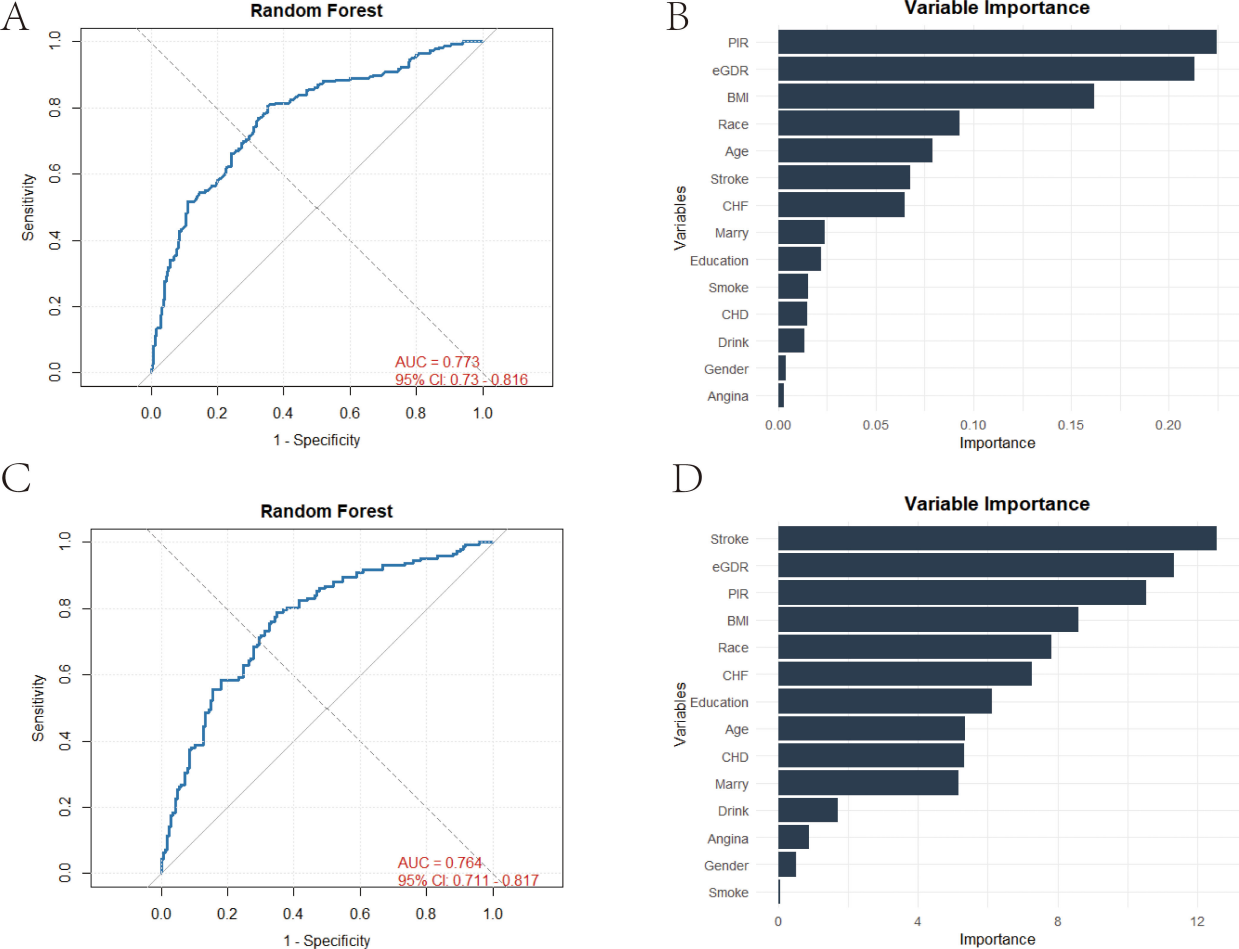
Receiver operating characteristic (ROC) curves and variable importance of XGBoost and random forest models for self-reported DR prevalence. **(A)** ROC curve of the XGBoost model. **(B)** Variable importance in the XGBoost model. **(C)** ROC curve of the random forest model. **(D)** Variable importance in the random forest model.

Then, we used XGBoost and the random forest algorithm to build a prediction model. As shown in **Figures 6A, C**, the AUC area of the XGBoost model is 0.773 (95% CI: 0.730–0.816), while the AUC of the random forest model is 0.764 (95% CI: 0.711–0.817). XGBoost is slightly better than random forests. Importantly, both models produce consistent rankings of variable importance and consistently identify eGDR, BMI, and PIR as the main contributors to DR prediction. These results further

**Figure 7:**
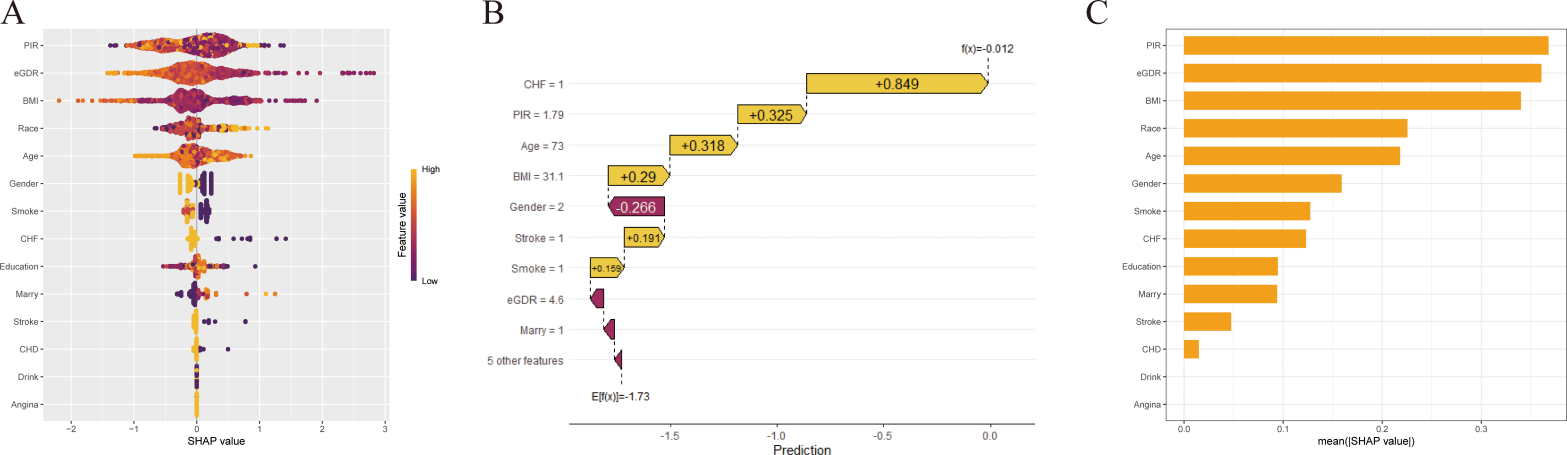
SHAP was applied to interpret the machine learning model for predicting self-reported DR prevalence. **(A)** SHAP summary plot showing the distribution and importance of each feature across participants, with colors indicating low (blue) to high (red) feature values. **(B)** SHAP force plot illustrating the contribution of individual features to the predicted DR risk for a representative participant. **(C)** Mean absolute SHAP values indicating the relative importance of features.

To enhance the interpretability of the model and clarify the directional impact of each predictor, we apply SHAP to the XGBoost model (**Figure 7**). The SHAP summary chart (**Figure 7A**) shows the distribution of contributions from all participants’ characteristics, indicating that PIR, eGDR, and BMI have the greatest impact on model predictions. It is worth noting that the lower eGDR value (shown in blue) is mainly associated with a positive SHAP value, indicating a close relationship between reduced eGDR and increased risk of diabetic retinopathy (DR).

**Figure 7B** shows the SHAP contributions of individual participants, visually depicting how specific characteristics jointly affect the predicted DR risk. In this case, lower eGDR, higher BMI, and older age are the main positive contributors (SHAP > 0), which together make the predictions tend to high risk. Consistent with this, the variables are sorted according to the average absolute SHAP value (average |SHAP|) (**Figure 7C**), which shows that PIR, eGDR, and BMI are the most influential predictors and the main drivers of model output.

## 4 Discussion

In this large-scale cross-sectional study using NHANES data, we observed a significant negative correlation between eGDR and the prevalence of self-reported DR. Specifically, the lower eGDR value is consistently associated with a higher prevalence of self-reported DR. To further assess the robustness of this association, we conducted a hierarchical analysis across multiple demographic and behavioral subgroups. It is worth noting that the negative correlation between eGDR and the self-reported DR prevalence is more significant in women and non-alcoholics, indicating that gender and lifestyle factors may have a regulatory effect on this association.

Insulin resistance is usually manifested as impaired cellular response to insulin, often accompanied by compensatory hyperinsulinemia and metabolic disorders, which play a central role in the pathogenesis of diabetic microvascular complications (28). The increased insulin resistance index (such as HOMA-IR) reflects a pathological metabolic environment that promotes chronic low-grade inflammation, oxidative stress, and endothelial dysfunction in retinal micro vessels and synergizes with persistent hyperglycemia (29, 30). This harmful environment will eventually accelerate pathological angiogenesis, including overexpression of VEGF, increased microvascular permeability, and capillary occlusion, thereby driving the occurrence and progression of DR (31, 32).

Increasing evidence supports the view that insulin resistance is an independent and early risk factor for DR (18, 33). Previous epidemiological and mechanistic studies have consistently shown a close relationship between insulin resistance and retinal microvascular damage (8, 34). For example, Zheng et al. reported that insulin signaling is crucial for retinal cell survival, and that increased insulin resistance is associated with early retinal microvascular abnormalities (which have been considered a precursor to DR), suggesting that insulin resistance may be a primary pathogenic driver (20). Similarly, Rowe et al. found that in patients with type 1 diabetes, the higher the level of insulin resistance, the higher the severity of DR (35). In summary, these findings lay a solid biological and clinical basis for the association between insulin resistance and DR (36).

Although hyperinsulinemia-normal blood glucose clamp technology remains the gold standard for quantifying insulin resistance, its invasiveness (37, 38), technical complexity, and time-consuming nature limit its widespread application in large-scale clinical and epidemiological research (39, 40). In this case, eGDR has become a practical and reliable indicator of insulin resistance (41, 42). Significantly, eGDR is closely related to insulin sensitivity assessed by the standard blood glucose clamp method, supporting its effectiveness as an indirect indicator of insulin-mediated glucose disposal (43). Our research results further expand the scope of eGDR’s application and highlight its strong correlation with DR risk in representative populations, thereby enhancing its potential value for early risk stratification and prevention strategies in clinical practice (42, 44).

Insulin resistance, reliably quantified by the eGDR, contributes to the onset and progression of DR through multiple interconnected pathophysiological pathways. It triggers chronic, low-grade inflammation by activating pro-inflammatory cytokines (TNF-α, IL-6, IL-1β), NF-κB signaling, and the NLRP3 inflammasome (45, 46). It also induces oxidative stress characterized by excessive reactive oxygen species (ROS) production and mitochondrial dysfunction (47, 48). Furthermore, insulin resistance impairs endothelial function by reducing nitric oxide (NO) bioavailability and upregulating endothelin-1 (ET-1) expression, activating the advanced glycation product (AGE)-receptor for AGE (RAGE) axis, and disrupting the balance between pro- and anti-angiogenic factors, leading to vascular endothelial growth factor (VEGF)-mediated pathological neovascularization (49). Collectively, these processes result in blood-retinal barrier breakdown, pericyte loss, and retinal microvascular damage. However, we must acknowledge that eGDR reflects glycemic control, adiposity, and hypertension, not insulin resistance alone.

### 4.1 Advantages and limitations

Although previous studies have examined the association between eGDR and DR prevalence, many were limited by relatively small sample sizes, selected clinical populations, or restricted analytical approaches. The present study utilized nationally representative data from the NHANES collected between 2007 and 2018, providing a large and diverse sample of U.S. adults. We comprehensively assessed the association between eGDR and self-reported DR prevalence, compared its performance with other insulin resistance-related indices, and incorporated interpretable machine learning approaches. These features extend the existing evidence by offering a broader population-based perspective on the relationship between insulin resistance and DR. Nevertheless, some limitations still need to be recognized. First, the cross-sectional nature of the study precludes causal inference, so the observed association between insulin resistance indicators and DR cannot be interpreted as a deterministic temporal correlation or causal effect. Therefore, prospective cohort research and mechanism research are needed to verify and expand these findings. Secondly, DR in this study was assessed based partly on self-reported data, which may be subject to recall bias and misclassification bias. Participants may inaccurately recall prior diagnoses, and true DR cases may be underreported or non-cases overreported. Consequently, the findings should be interpreted with caution and apply specifically to self-reported DR rather than clinically confirmed disease. Hence, future research should give priority to employing standardized fundus photography or evaluations conducted by ophthalmologists as the gold standard for DR diagnosis. Third, despite the extensive adjustment of covariables, the residual mixing factors cannot be eliminated. Specifically, the absence of certain variables (such as a detailed history of drug use or familial dyslipidemia tendency) may introduce unmeasured confounders. Lastly, owing to limitations in the NHANES data, we were unable to distinguish between type 1 and type 2 diabetes.

## 5 Conclusion

In conclusion, this nationally representative study demonstrates a significant inverse association between eGDR and self-reported DR prevalence. These findings indicate that lower eGDR levels correlate with higher self-reported DR prevalence. However, given the limitations of a cross-sectional study design, a definitive causal relationship cannot be established from the current data. Future large-scale prospective cohort studies encompassing diverse ethnic and demographic populations are warranted to validate these associations and assess their broader generalizability.

## Data Availability

The raw data supporting the conclusions of this article will be made available by the authors, without undue reservation.
