# Supplementary material for: The association between estimated glucose disposal rate and self-reported diabetic retinopathy: evidence from two independent cohorts and machine learning
Source: Front Endocrinol (Lausanne). 2026 Jun 26;17:1853329. doi: 10.3389/fendo.2026.1853329 (PMC13349794; doi:10.3389/fendo.2026.1853329)
Supplement: Supplementary file 1 [file Table1.docx]

Table S1. Specific calculation formulas for insulin resistance indices.

| Indicators | Calculation formula |
| --- | --- |
| HOMA-IR | fasting glucose (mmol/L) × fasting insulin (µU/mL)/22.5 |
| HOMA-IS | 22.5/fasting glucose (mmol/L) × fasting insulin (µU/mL) |
| HOMA-B | [20 × fasting insulin (uU/L)]/ [fasting glucose (mmol/L) −3.5] |
| METS-IR | Ln [2 × fasting glucose(mg/dL) + triglycerides(mg/dL)] × BMI/Ln HDL-C (mg/dL) |
| eGDR | 21.158 – [0.09 × waist circumference (WC)] − (3.407 × hypertension) − [0.551 × glycosylated hemoglobin (HbA1c)] |
| QUICKI | 1/ [log (fasting insulin(µU/mL)) + log (fasting plasma glucose(mg/dL)] |

Table S2. Detailed information on relevant covariates.

| Category | Variable | Classification | Data Source |
| --- | --- | --- | --- |
| Demographic Data | Age | <60 / ≥60 | Population statistics data |
|  | Gender | Male / Female |  |
|  | Ethnicity | Mexican American / Other Hispanic /  Non-Hispanic White / Non-Hispanic Black / Other |  |
|  | Education Level | Less Than 9th Grade / 9-11th Grade / High school graduate / Some college or AA degree / College graduate or above |  |
|  | Economic Level | PIR [<1, 1-3, >3] |  |
|  | Marital Status | Unmarried or other/ Married or living with a partner |  |
| Physical Examination | Body Mass Index (BMI) | <25, 25-30, >30 | Physical examination |
| Health-Related Behaviors | Smoking Status | Never smoked / Former smoker / now smoker |  |
|  | Drinking Status | Yes/No |  |
| Comorbidities | Hypertension | Yes/No | Questionnaire data |
|  | Coronary Heart Disease | Yes/No |  |
|  | Heart Failure | Yes/No |  |
|  | Stroke | Yes/No |  |
|  | Angina | Yes/No |  |

**Table S3.** Demographics classified with Quartiles of eGDR.

|  | All | Quartile 1 | Quartile 2 | Quartile 3 | Quartile 4 | p-value |
| --- | --- | --- | --- | --- | --- | --- |
| Number | 1536 | 384 | 384 | 384 | 384 |  |
| eGDR (mean (SD)) | 4.56(2.48) | 1.66 (1.17) | 3.78 (0.44) | 5.22 (0.46) | 7.91 (1.24) | <0.001 |
| Gender (N, %) |  |  |  |  |  | 0.025 |
| Male | 818(52.6) | 215(55.3) | 214(57.8) | 185(42.5) | 204(53.9) |  |
| Female | 718(47.4) | 169(44.7) | 170(42.2) | 199(57.5) | 180(46.1) |  |
| Age [years, mean (SD)] | 59.84(12.96) | 57.54(11.42) | 62.67(11.53) | 61.49(12.97) | 57.89(15.04) | <0.001 |
| Ethnicity (N, %) |  |  |  |  |  | 0.038 |
| Mexican American | 263(8.8) | 40(5.4) | 66(7.8) | 77(10.3) | 80(12.0) |  |
| Other Hispanic | 185(6.3) | 49(6.7) | 41(5.5) | 40(4.9) | 55(8.1) |  |
| Non-Hispanic White | 575(64.4) | 155(65.4) | 147(66.8) | 138(64.5) | 135(60.7) |  |
| Non-Hispanic Black | 366(13.0) | 121(16.3) | 104(14.1) | 85(12.6) | 56(8.8) |  |
| Other | 147(7.5) | 19(6.2) | 26(5.8) | 44(7.8) | 58(10.4) |  |
| Education (N, %) |  |  |  |  |  | 0.166 |
| Less Than 9th Grade | 240(9.1) | 53(8.4) | 67(11.5) | 66(9.0) | 54(7.6) |  |
| 9-11th Grade | 271(13.7) | 68(11.6) | 68(12.7) | 73(16.9) | 62(14.0) |  |
| Highschool graduate or equivalent | 339(24.9) | 85(26.7) | 70(19.2) | 92(29.2) | 92(24.6) |  |
| Some Colleges or AA degree | 429(30.9) | 120(34.6) | 112(32.4) | 100(28.4) | 97(27.5) |  |
| College graduate or above | 257(21.4) | 58 (18.7) | 67(24.2) | 53(16.4) | 79(26.2) |  |
| BMI |  |  |  |  |  | <0.001 |
| <25 | 194(10.4) | 4(0.5) | 14(3.8) | 55(11.8) | 121(26.9) |  |
| [25,30] | 467(27.5) | 30 (5.8) | 90(20.3) | 192(45.1) | 155(42.4) |  |
| >30 | 875(62.0) | 350(93.7) | 280(75.9) | 137(43.1) | 108(30.7) |  |
| Economic level (N, %) |  |  |  |  |  | 0.571 |
| <1 | 341(14.9) | 90(15.8) | 81(12.9) | 84(15.1) | 86(15.8) |  |
| 1-3 | 734(42.9) | 177(42.7) | 181(41.7) | 204(48.0) | 172(39.6) |  |
| >3 | 461(42.2) | 117(41.5) | 122(45.4) | 96(36.9) | 126(44.6) |  |
| Marital status (N, %) |  |  |  |  |  | 0.800 |
| Unmarried or other | 614(35.6) | 165(38.0) | 158(34.4) | 140(34.0) | 151(35.6) |  |
| Married or living with a partner | 922(64.4) | 219(62.0) | 226(65.6) | 244(66.0) | 233(64.4) |  |
| Alcohol consumption (N, %) |  |  |  |  |  | 0.630 |
| Yes | 1077(71.9) | 272(73.6) | 270(74.1) | 266(69.4) | 269(70.0) |  |
| No | 459(28.1) | 112(26.4) | 114(25.9) | 118(30.6) | 115(30.0) |  |
| Smoking status (N, %) |  |  |  |  |  | 0.373 |
| Never | 767(50.2) | 182(47.9) | 190(49.2) | 193(50.5) | 202(53.2) |  |
| Now | 237(14.5) | 55(13.9) | 51(11.1) | 66(17.4) | 65(15.7) |  |
| Former | 532(35.4) | 147(38.2) | 143(39.6) | 125(32.1) | 117(31.1) |  |
| Hypertension (N, %) |  |  |  |  |  | <0.001 |
| Yes | 1143(72.6) | 374(97.2) | 368(94.3) | 321(79.7) | 80(16.6) |  |
| No | 393(27.4) | 10(2.8) | 16(5.7) | 63(20.3) | 304(83.4) |  |
| CHD (N, %) |  |  |  |  |  | 0.065 |
| Yes | 175(11.4) | 44(12.1) | 51(12.5) | 54(15.6) | 26(5.5) |  |
| No | 1361(88.6) | 340(87.9) | 333(87.5) | 330(84.4) | 358(94.5) |  |
| CHF (N, %) |  |  |  |  |  | 0.074 |
| Yes | 143(8.7) | 39(10.4) | 49(11.0) | 34(8.6) | 21(4.5) |  |
| No | 1393(91.3) | 345(89.6) | 335(89.0) | 350(91.4) | 363(95.5) |  |
| Angina (N, %) |  |  |  |  |  | 0.045 |
| Yes | 107(7.4) | 29(6.8) | 40(11.8) | 28(8.2) | 10(3.0) |  |
| No | 1429(92.6) | 355(93.2) | 344(88.2) | 356(91.8) | 374(97.0) |  |
| Stroke (N, %) |  |  |  |  |  | 0.632 |
| Yes | 139(8.0) | 32(9.2) | 48(8.8) | 32(6.2) | 27(7.5) |  |
| No | 1397(92.0) | 352(90.8) | 336(91.2) | 352(93.8) | 357(92.5) |  |
| HOMA_IR (mean (SD)) | 10.72(14.53) | 15.41(17.82) | 9.75(10.27) | 8.93 (13.96) | 8.28(13.65) | <0.001 |
| HOMA_IS (mean (SD)) | 0.28(0.62) | 0.15(0.28) | 0.24(0.29) | 0.30(0.38) | 0.46(1.09) | <0.001 |
| HOMA_B (mean (SD)) | 137.35(495.60) | 127.16(215.92) | 133.18(167.74) | 141.34(927.46) | 149.09(339.77) | 0.783 |
| METS_IR (mean (SD)) | 57.45(18.51) | 69.10(15.94) | 55.60 (13.04) | 54.03(20.42) | 49.78(18.11) | <0.001 |
| QUICKI (mean (SD)) | 2.88(3.00) | 3.05(0.34) | 3.04(0.32) | 2.42(5.95) | 2.93(1.72) | 0.631 |

**Notes: Continuous variables are presented as weighted means (standard Deviation). Categorical variables are presented as unweighted counts (weighted percentages).**

**Abbreviations:** DR: diabetic retinopathy; SD: Standard Deviation; CHD: Coronary heart disease; CHF: congestive heart failure

Table S4. Sensitivity Analysis of eGDR and self-reported DR prevalence After Excluding Participants with eGDR <2 or ≥8

|  | Model 1 | *P*-value | Model 2 | *P*-value | Model 3 | *P*-value |
| --- | --- | --- | --- | --- | --- | --- |
| eGDR continuous | 0.88(0.74,1.05) | 0.1641 | 0.88(0.74,1.06) | 0.1690 | 0.76(0.60,0.97) | 0.0250 |
| eGDR quartile |  |  |  |  |  |  |
| Q1 | Reference |  | Reference |  | Reference |  |
| Q2 | 0.52(0.22,1.20) | 0.1211 | 0.51(0.22,1.19) | 0.1157 | 0.41(0.18,0.90) | 0.0273 |
| Q3 | 0.68(0.29,1.58) | 0.3684 | 0.67(0.27,1.64) | 0.3727 | 0.45(0.18,1.11) | 0.0811 |
| Q4 | 0.49(0.22,1.08) | 0.0758 | 0.48(0.21,1.07) | 0.0707 | 0.32(0.12,0.83) | 0.0200 |

Model 1: unadjusted.

Model 2: Model 1 + sex, age, and ethnicity.

Model 3: Model 2 + educational level, marital status, economic level, BMI, alcohol consumption, smoking status, hypertension, CHF, CHD, angina, and stroke.

Abbreviations: BMI: body mass index; OR: odds ratio; CI: confidence interval; eGDR: estimated glucose disposal rate; DR: diabetic retinopathy.

Table S5. Sensitivity Analysis of eGDR and self-reported DR prevalence using unweighted data.

|  | Model 1 | *P*-value | Model 2 | *P*-value | Model 3 | *P*-value |
| --- | --- | --- | --- | --- | --- | --- |
| eGDR continuous | 0.90(0.85,0.95) | <0.001 | 0.89(0.84,0.94) | <0.0001 | 0.77(0.60,0.97) | <0.0001 |
| eGDR quartile |  |  |  |  |  |  |
| Q1 | Reference |  | Reference |  | Reference |  |
| Q2 | 0.78(0.55,1.09) | 0.1441 | 0.76(0.54,1.07) | 0.1203 | 0.61(0.69,0.86) | 0.0124 |
| Q3 | 0.65(0.46,0.92) | 0.0166 | 0.62(0.43,0.90) | 0.0112 | 0.46(0.29,0.72) | 0.0007 |
| Q4 | 0.53(0.37,0.76) | 0.0006 | 0.50(0.34,0.73) | 0.0003 | 0.35(0.19,0.67) | 0.0016 |

Model 1: unadjusted.

Model 2: Model 1 + sex, age, and ethnicity.

Model 3: Model 2 + educational level, marital status, economic level, BMI, alcohol consumption, smoking status, hypertension, CHF, CHD, angina, and stroke.

Abbreviations: BMI: body mass index; OR: odds ratio; CI: confidence interval; eGDR: estimated glucose disposal rate; DR: diabetic retinopathy. CHF:

Table S6. Variance inflation factor (VIF) values for the multicollinearity diagnosis of independent variable

| Variables | VIF |
| --- | --- |
| Age | 1.963190 |
| Gender | 1.418399 |
| Race | 1.340603 |
| Education | 1.335429 |
| Economic level | 1.460004 |
| Marry status | 1.265423 |
| Smoke | 1.443029 |
| Drink | 1.312427 |
| Hypertension | 2.196897 |
| CHF | 1.390448 |
| CHD | 1.569555 |
| Angina | 1.562303 |
| Stroke | 1.389615 |
| BMI | 1.832494 |

Abbreviations: CHD: Coronary heart disease; CHF: congestive heart failure. BMI: body mass index.

| **variable** | **Model** | |
| --- | --- | --- |
|  | **OR (95%CI)** | ***p-*value** |
| **eGDR (continuous)** | 0.85(0.77, 0.94) | 0.0029 |

Table S7. Sensitivity Analysis of eGDR and self-reported DR prevalence after excluding the component variables of eGDR

The model was adjusted: age, sex, ethnicity, educational level, marital status, economic level, BMI, alcohol consumption, smoking status, CHF, CHD, angina, and stroke.

**Table S8.** Differences in Baseline Characteristics Between Included and Excluded Participants.

|  | Exclude | Include | *p-value* |
| --- | --- | --- | --- |
| Number | 4422 | 1536 |  |
| **Age [years, mean (SD)]** | 58.51 (15.22) | 59.84 (12.96) | 0.007 |
| **Gender (N, %)** |  |  | 0.273 |
| Male | 2207(50.2) | 818(52.6) |  |
| Female | 2215(49.8) | 718(47.4) |  |
| **Education (N, %)** |  |  | 0.643 |
| Less Than 9th Grade | 829(10.8) | 240(9.1) |  |
| 9-11th Grade | 724(13.2) | 271(13.7) |  |
| Highschool graduate or equivalent | 1017(24.9) | 339(24.9) |  |
| Some Colleges or AA degree | 1157(31.1) | 429(30.9) |  |
| College graduate or above | 695(20.0) | 257(21.4) |  |
| **Marital status (N, %)** |  |  | 0.393 |
| Unmarried or other | 1855(37.4) | 614(35.6) |  |
| Married or living with a partner | 2567(62.6) | 922(64.4) |  |
| **Economic level (N, %)** |  |  | 0.096 |
| <1 | 1141(18.5) | 341(14.9) |  |
| 1-3 | 2054(41.9) | 734(42.9) |  |
| >3 | 1227(39.7) | 461(42.2) |  |
| **Ethnicity (N, %)** |  |  | <0.001 |
| Mexican American | 798(10.4) | 263(8.8) |  |
| Other Hispanic | 469(5.8) | 185(6.3) |  |
| Non-Hispanic White | 1369(56.8) | 575(64.4) |  |
| Non-Hispanic Black | 1227(16.7) | 366(13.0) |  |
| Other | 559(10.4) | 147(7.5) |  |
| **Alcohol consumption (N, %)** |  |  | 0.830 |
| Yes | 2932(71.4) | 1077(71.9) |  |
| No | 1483(28.6) | 459(28.1) |  |
| **CHD (N, %)** |  |  | 0.784 |
| Yes | 441(11.1) | 175(11.4) |  |
| No | 3981(88.9) | 1361(88.6) |  |
| **Angina (N, %)** |  |  | 0.877 |
| Yes | 299(7.6) | 107(7.4) |  |
| No | 4123(92.4) | 1429(92.6) |  |
| **Stroke (N, %)** |  |  | 0.798 |
| Yes | 396(8.3) | 139(8.0) |  |
| No | 4026(91.7) | 1397(92.0) |  |
| **BMI (kg/m^2^)** |  |  | 0.218 |
| <25 | 599(11.8) | 194(9.1) |  |
| [25,30] | 1228(13.2) | 467(13.7) |  |
| >30 | 2595(24.9) | 875(24.9) |  |
| **Smoking status (N, %)** |  |  | 0.473 |
| Never | 2275(49.2) | 767(50.2) |  |
| Now | 707(16.4) | 237(14.5) |  |
| Former | 1440(34.4) | 532(35.4) |  |
| **CHF (N, %)** |  |  | 0.837 |
| Yes | 430(8.9) | 143(8.7) |  |
| No | 3992(91.1) | 1393(91.3) |  |

**Notes: Continuous variables are presented as weighted means (standard Deviation). Categorical variables are presented as unweighted counts (weighted percentages).**

**Abbreviations:** SD: Standard Deviation; CHD: Coronary heart disease; CHF: congestive heart failure; BMI: body mass index.
